# The relationship between geochemical background values of MgO and SiO₂ and regional population longevity: evidence from Yunnan, China

**DOI:** 10.3389/fpubh.2025.1595130

**Published:** 2025-07-02

**Authors:** Xin Zhou, Jiaxue Wang, Shuangshuang Zhu, Min Lai

**Affiliations:** Faculty of Geography, Yunnan Normal University, Kunming, China

**Keywords:** SiO_2_, population longevity level, correlation analysis, MgO, geochemical background value

## Abstract

**Introduction:**

The relationship between geographical environments and human health has been a long-standing focus of scientific inquiry. Magnesium (Mg) and silicon (Si), as essential elements for the human body, play vital roles in individual health and may influence longevity. However, the extent to which the statistical characteristics of population longevity are associated with geochemical background values at a regional scale remains an important question.

**Methods:**

This study examines Yunnan, China, a region with diverse and complex geographical conditions, and used global autocorrelation analysis, cluster and outlier analysis, and hotspot analysis to comprehensively analyze the Spatial Distribution Characteristics of magnesium oxide (MgO) and silicon dioxide (SiO₂) background values. It further investigates the individual and synergistic relationships of these geochemical factors with population longevity at the county scale in Yunnan using the Spearman rank correlation.

**Results:**

The results demonstrate that the MgO background value (ω(MgO)) exhibits a significant positive correlation with the Ultra-octogenarian Index and has a positive synergistic effect on regional longevity levels. In contrast, the SiO₂ background value (ω(SiO₂)) shows a significant negative correlation with both the longevity index and the Ultra-octogenarian Index, while the ratio of Si to Mg (ω(Si/Mg)) is also significantly negatively correlated with the Ultra-octogenarian Index.

**Discussion:**

These findings suggest that MgO-enriched natural environments may positively contribute to regional population longevity, while excessively high SiO₂ background values may have a detrimental effect. This study offers a novel perspective on the relationship between regional longevity levels and natural geographical environments, which may inform the selection and sustainable development of longevity-oriented tourism destinations.

## 1 Introduction

Health, longevity, physical fitness, and mental well-being have become universal goals for humanity (1). Increasing attention has been given to the factors influencing human health and longevity, with the aim of improving overall life expectancy. Genetic, environmental (such as climate, water quality, and soil composition), and socioeconomic factors (including lifestyle, healthcare, and economic conditions) all play a role in shaping human health and longevity to varying extents (2–5). In recent years, the impact of natural environmental factors on human health and longevity has garnered widespread interest within the geoscientific community. Significant research findings have emerged from case studies conducted in various regions of China. For example, excessive exposure to elements such as chromium (Cr), cadmium (Cd), and lead (Pb) in the environment has been linked to increased cancer risks (6–8). Environmental exposure to thallium can cause endemic thallium (TI) poisoning, which adversely affects the growth and development of fetuses and children (9). In the selenium-deficient belt extending from northeastern Heilongjiang to the southwestern Tibetan Plateau, selenium deficiency has been associated with diseases such as Keshan disease and Kashin-Beck disease in regions like Keshan County, Heilongjiang, and Chuxiong Prefecture, Yunnan (10–12). In longevity areas such as the Xicheng District of Beijing and the Chéngmài Longevity Village in Hainan Province, trace element profiles of older adult individuals show a striking similarity to the local soil element composition (13, 14). In Xiayi County, Henan Province, the uneven distribution of selenium (Se), zinc (Zn), copper (Cu), and sodium (Na) in the soil is identified as a key factor influencing the distribution of long-lived populations in the region (15). In Bama, Guangxi Province, the presence of elements like zinc (Zn) and manganese (Mn) in the local well water is considered crucial for the longevity of its residents (16). In Shandong Province, the relatively high levels of selenium (Se) and strontium (Sr) in the groundwater of longevity towns such as Chengyang, Rushan, Pingyi, and Shan County have been identified as one of the main factors contributing to the longevity of their populations (17).

These studies provide valuable data and insights into the relationship between individual health, longevity, and geochemical elements. However, the potential correlation between regional human health and longevity statistics and the geochemical background values remains an intriguing scientific question.

Magnesium (Mg) and silicon (Si) are essential elements for the human body. The human body contains approximately 35 g of magnesium, which plays a critical role in activating enzyme activity, maintaining the stability of ribonucleic acids and nucleoproteins, and is indispensable in physiological metabolic processes (18). Silicon, on the other hand, participates in the process of bone calcification, benefits cartilage and connective tissues, protects the cardiovascular system, and influences the aging process, making it an essential trace element for the human body (19). Both magnesium and silicon predominantly exist in the environment as oxides, namely magnesium oxide (MgO) and silicon dioxide (SiO_2_). These elements are abundant in water system sediments and soils and are chemically active. Studies have indicated that both elements significantly affect soil fertility and plant nutrition, thereby indirectly influencing human health (20). However, the specific impact of MgO and SiO_2_ on human longevity remains unclear, both at the individual and regional scales.

Located on the southeastern edge of the Tibetan Plateau and the central part of the Yunnan-Guizhou Plateau, Yunnan Province is characterized by climatic diversity, geological and geomorphological variety, diverse soil types, and rich biodiversity. This study focuses on Yunnan Province to explore the correlation between the geochemical background values of MgO and SiO_2_ and the statistical characteristics of human longevity at the county level. The aim is to examine whether distinct spatial statistical patterns persist under the influence of various complex factors. Based on this understanding, the study utilizes ArcGIS for spatial analysis, SPSS for statistical methods, MATLAB programming, and GeoDa spatial data analysis software to visually analyze the spatial differentiation of MgO and SiO_2_ geochemical background values and the distribution of human longevity across counties in Yunnan. The study also seeks to identify potential correlations, providing valuable insights for further understanding the relationship between magnesium, silicon, and human longevity in the context of geographical environments.

## 2 Materials and methods

### 2.1 Overview of the study area

**Figure 1:**
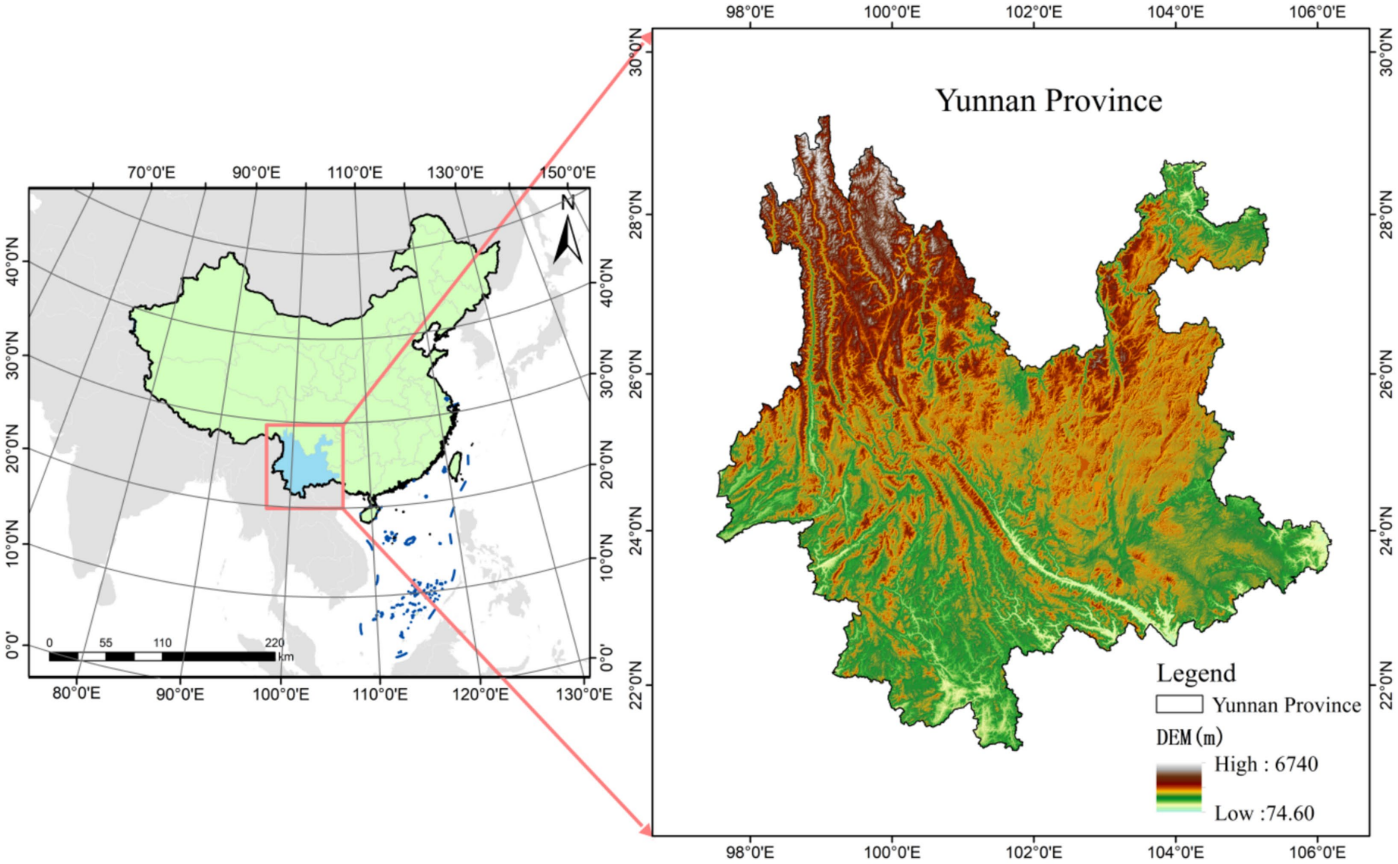
Overview map of the study area.

Yunnan Province (21°8′–29°15′N, 97°31′–106°11′E), located in the southwest of China, spans across the Tropic of Cancer in its central and southern regions, positioning it as a low-latitude inland area, as depicted in Figure 1. The province is characterized by a mountainous plateau terrain, with the elevation decreasing in a stepped manner from north to south, exhibiting a pattern of higher elevations in the northwest and lower elevations in the southeast. The western and northwestern parts of Yunnan are an extension of the Tibetan Plateau, featuring major mountain ranges such as the Gaoligong, Nu, and Yunling Mountains, and large rivers, including the Nujiang, Lancang, and Jinsha Rivers, which flow from north to south in an alternating manner. The southern part of the province is dominated by the Hengduan Mountain range, including prominent mountains like the Ailao, Wuliang, and Bangma ranges, with the terrain gently sloping toward the southwest and river valleys gradually widening. The climate of Yunnan is diverse, encompassing three climatic zones: cold, temperate, and hot (including subtropical), with temperature variations closely associated with elevation. This results in a distinct vertical climatic profile. The northwestern region of Yunnan experiences a cold climate, while the eastern and central areas are temperate, and the southern and southwestern regions fall within low-heat river valleys. Some areas, situated south of the Tropic of Cancer, extend into the tropical zone. The average temperature in the hottest month (July) ranges from 19°C to 22°C, while in the coldest month (January), the average temperature exceeds 6°C to 8°C. The annual temperature difference typically ranges from 10°C to 12°C. Yunnan is also characterized by significant volcanic activity, which has persisted within the same region, forming large tectonic magma belts and various types of igneous rocks. Moreover, the complex interplay of factors such as climate, biology, topography, parent material, and vegetation has led to the development of a wide range of soil types, with particularly pronounced vertical soil distribution patterns. In terms of soil classification, Yunnan’s soils can be categorized into 7 soil orders, 19 soil classes, 34 sub-classes, 145 soil genera, and 288 cultivated soil types. Common soil types include red soils, yellow-red soils, yellow-brown soils, brown soils, dark brown soils, lime (rock) soils, and purple soils (21). The mineral composition of these soils varies significantly due to differences in parent material and environmental conditions. Furthermore, the diverse land-use practices, influenced by the livelihoods of various ethnic groups, have contributed to considerable variations in the chemical composition of soil elements across different regions.

As of the end of 2023, Yunnan Province is composed of 8 prefecture-level cities, 8 autonomous prefectures, and 129 county-level administrative districts, which include 17 urban districts, 18 county-level cities, 65 counties, and 29 ethnic autonomous counties. By the close of 2023, the province’s resident population totaled 46.73 million, with 24.73 million living in urban areas and 22. 00 million in rural areas, resulting in an urbanization rate of 52.92%. In the same year, there were 385,000 births, yielding a birth rate of 8.22‰; 403,000 deaths, corresponding to a death rate of 8. 61‰; and a natural population growth rate of −0.39‰.

### 2.2 Data source and preprocessing

#### 2.2.1 Element background value data

The elemental data used in this study are geochemical background values. The MgO and SiO_2_ background values for each county-level administrative region in Yunnan Province are sourced from the Geophysical and Geochemical Atlas of Yunnan Province. This atlas was compiled by the Institute of Physical and Chemical Exploration of the Yunnan Geological Survey Bureau, based on data collected over 20 years (from 1979) through regional gravity surveys at a scale of 1:200,000, small-scale aeromagnetic surveys, and 1:200,000 regional geochemical surveys. The geochemical mapping utilized water system sediments as the sampling medium (with soil used as a substitute in areas without water system sediments), with a sampling density of 1–2 samples per km^2^, resulting in a total of 96,399 samples (22). Prior to 2000, resource development in Yunnan was relatively low, and human interference was minimal. The regional exploration data from 1979 to 1999 closely align with the regional background values, making this the most comprehensive and reliable dataset currently available for MgO and SiO_2_ distribution. Therefore, this study uses the geochemical maps of magnesium oxide (MgO) and silicon dioxide (SiO_2_) in Yunnan Province as the base map. Using the ArcGIS 10.8 platform, the images were vectorized, and topological checks and attribute data verification were performed on the vectorized results. Any issues encountered were addressed through technical processing to ensure the reliability of the data quality. In this study, *ω*(MgO) and *ω*(SiO_2_) represent the geochemical background values of MgO and SiO_2_ at the regional level.

#### 2.2.2 Longevity indicators and population data

Currently, commonly used longevity indicators include the centenarian ratio (CR, the number of centenarians per 100,000 people), longevity index (LI‰, the ratio of people aged 90 and above to those aged 65 and above, expressed per thousand), the Ultra-octogenarian Index (UOI%, the percentage of people aged 80 and above in the population aged 60 and above), life expectancy (LE, the average number of years a population born in a specific year is expected to live), and modal age of death (the age at which most adult deaths occur) (23–29). However, the applicability of these indicators varies. The centenarian ratio is the most widely recognized and universally accepted measure, but it is susceptible to extreme values in populations under 500,000 (30, 31). Life expectancy is frequently used to assess differences in longevity across regions or populations, but it is heavily influenced by economic development and healthcare levels, making it more useful for understanding the relationship between socioeconomic factors and longevity (32, 33). The modal age of death provides a unique perspective on regional longevity by reflecting the concentrated age group of deaths, but may not accurately reflect local health levels in small or unstable datasets (29). In contrast, the longevity index is less affected by other factors and provides a more accurate reflection of longevity at the county and township scale. The Ultra-octogenarian Index provides an intuitive representation of a region’s older adult population, facilitating comparisons between different areas (31, 34).

Given that 104 out of 129 counties in Yunnan Province have populations under 500,000, this study uses the longevity index and the Ultra-octogenarian Index as longevity indicators due to their greater stability and applicability, providing a more objective measure of regional longevity levels. Considering that the influence of geochemical elements on regional longevity is accumulated over time, and to exclude the impact of COVID-19 on the longevity population, the data used in this study are derived from the Seventh National Census of China, conducted in 2020. This dataset includes the total county population and age-specific population counts.

### 2.3 Research methods

#### 2.3.1 Global autocorrelation analysis

Global spatial autocorrelation reflects the overall distribution of a phenomenon, analyzing whether clustering characteristics exist in its spatial distribution (35, 36). This study employs global spatial autocorrelation analysis to investigate the clustering patterns of ω(oxide) at the county scale in Yunnan Province. The analysis uses Moran’s I index as the statistical metric, and its formula is as shown in Equation 1:


(1) $$I=\frac{n\cdot \sum_{i=1}^{n}\sum_{j=1}^{n}w_{\text{ij}}(x_{i}-x)(x_{j}-x)}{(\sum_{i=1}^{n}\sum_{j=1}^{n}w_{\text{ij}})\cdot \sum_{i=1}^{n}{\left(x_{i}-x\right)}^{2}},i\neq j$$

In the formula, *I* represents the Moran’s *I* index; *n* denotes the total number of study units, i.e., the total number of counties; $x_{i}\;$and $x_{j}$ are the attribute values of the *i*-th and *j*-th regions, respectively, representing the *ω*(oxide) values of the counties; $\bar{x}$ is the mean of all regional attribute values, i.e., the average *ω*(oxide) value across all counties; $w_{\text{ij}}\;$is the spatial weight matrix, which represents the adjacency relationships of the spatial objects. The Moran’s *I* index ranges between [−1, 1]:

*I* > 0: indicates positive spatial autocorrelation, meaning *ω*(oxide) exhibits clustering in its spatial distribution.
*I* = 0: indicates a random spatial distribution.
*I* < 0: indicates negative spatial autocorrelation, meaning *ω*(oxide) is dispersed in its spatial distribution Equation 2.

The standardized test statistic *Z* is used to evaluate the significance of Moran’s I index, and its formula is as shown in Equation 2:


(2) $$Z=\frac{1-E(I)}{\sqrt{\text{VAR}(I)}}=\frac{\sum_{j\neq 1}^{n}W_{\text{ij}}(d)(x_{j}-{\bar{x}}_{i})}{S_{i}\sqrt{\frac{w_{i}(n-1-w_{i})}{n-2}}},(i\neq j)$$

#### 2.3.2 Cluster and outlier analysis

Cluster and outlier analysis tools can identify spatial clusters with high or low-value features as well as spatial outliers. Using the Local Moran’s I index, spatial clustering patterns of *ω*(oxide) with relatively high or low values at the county scale can be detected (36, 37). The formula is as shown in Equation 3:


(3) $$I_{i}=\frac{x_{i}-x}{\frac{\sum_{i=1,j\neq 1}^{n}{\left(x_{j}-x\right)}^{2}}{n-1}}\sum_{i=1,j\neq 1}^{n}\omega_{i,j}(x_{j}-x)$$

The statistical score is calculated according to Equation 4:


(4) $$Z_{I_{i}}=\frac{I_{i}-E[I_{i}]}{\sqrt{\left(E[I_{i}^{2}]-E{\left[I_{i}\right]}^{2}\right)}}$$

Of which E[I_i_] is as shown in Equation 5:


(5) $$E[I_{i}]=-\frac{\sum_{j=1,j\neq i}^{n}w_{\text{ij}}}{n-1}$$

In the formula, $x_{i}\;$ and $x_{j}\;$represent the observed values of a phenomenon in spatial units i and j, respectively. The meanings of the remaining symbols are the same as those in Equation 1. A positive *I* value indicates that the feature is part of a cluster, meaning the feature has neighboring features with similarly high or low attribute values. A negative *I* value indicates that the feature is an outlier, meaning the feature has neighboring features with contrasting attribute values.

Using the Cluster and Outlier Analysis tool in ArcGIS 10.8, the spatial distribution of *ω*(oxide) at the county level in Yunnan Province is analyzed. The significance of the Local Moran’s *I* index is assessed through its value, Z-score, *p*-value, and the cluster type code assigned to each feature. The analysis identifies two types of clustering patterns: High-High (HH) clusters, where high values are surrounded by similarly high values, and Low-Low (LL) clusters, where low values are surrounded by similarly low values. Additionally, two types of outliers are identified: Low-High (LH), where low values are surrounded by high values, and High-Low (HL), where high values are surrounded by low values.

#### 2.3.3 Hotspot analysis

Hotspot analysis is a spatial statistical method used to identify areas with significant clustering of high or low values. It provides a deeper understanding of the spatial distribution and intensity of a given phenomenon (38). In this study, the Getis-statistic is employed to conduct hotspot analysis at the county scale, exploring the spatial distribution of ω(oxide) in Yunnan Province. The formula is as shown in Equation 6:


(6) $$G_{i}^{*}=\frac{\sum_{j=1}^{n}w_{i,j}x_{j}-x\sum_{j=1}^{n}w_{i,j}}{\sqrt{\frac{\sum_{j=1}^{n}x_{j}^{2}}{n}-x^{2}}\sqrt{\frac{n\sum_{j=1}^{n}w_{i,j}^{2}-{\left(\sum_{j=1}^{n}w_{i,j}\right)}^{2}}{n-1}}\;}$$

In the formula, the symbolic meaning is the same as before. The statistical significance of the Getis-$G_{i}^{*}$ values is evaluated using Z-scores and *p*-values. The results identify hotspots (clusters of high *ω* values) and coldspots (clusters of low *ω* values).

#### 2.3.4 Spearman rank correlation

The Spearman rank correlation coefficient is a non-parametric measure of the strength of the monotonic relationship between two variables. Unlike the Pearson correlation coefficient, it does not require the data to follow a normal distribution. The Spearman correlation coefficient, denoted as $r_{s}$, ranges from [−1, 1], with larger absolute values indicating stronger correlation. A value of −1 represents perfect negative correlation, 1 represents perfect positive correlation, and 0 indicates no monotonic relationship (39). The formula is as shown in Equation 7:


(7) $$r_{s}=1-\frac{6\sum_{i=1}^{N}d_{i}^{2}}{N(N^{2}-1)}$$

In the formula, *r_s_* is the Spearman correlation coefficient, *d_i_* is the rank difference between the *i*-th observations of the two variables (i.e., the difference between the ranks of *Xi* and *Yi*, where *X* and *Y* are the observed values of the two variables), and *N* is the sample size.

## 3 Results

### 3.1 Spatial distribution characteristics of MgO and SiO_2_

#### 3.1.1 Global autocorrelation of *ω*(MgO) and *ω*(SiO_2_)

**Figure 2:**
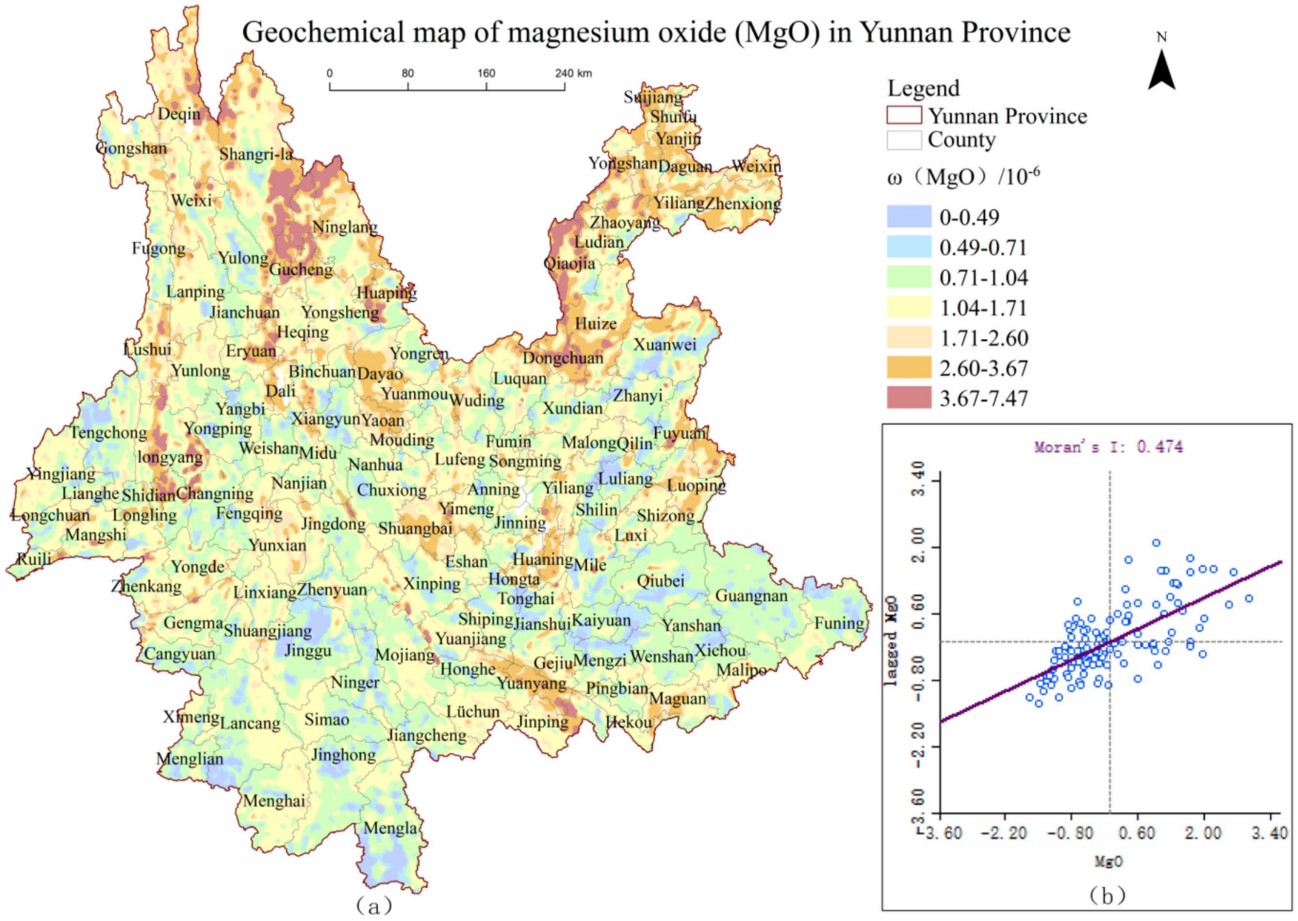
Spatial distribution pattern of *ω*(MgO) and global autocorrelation scatter plot in Yunnan Province.

**Figure 3:**
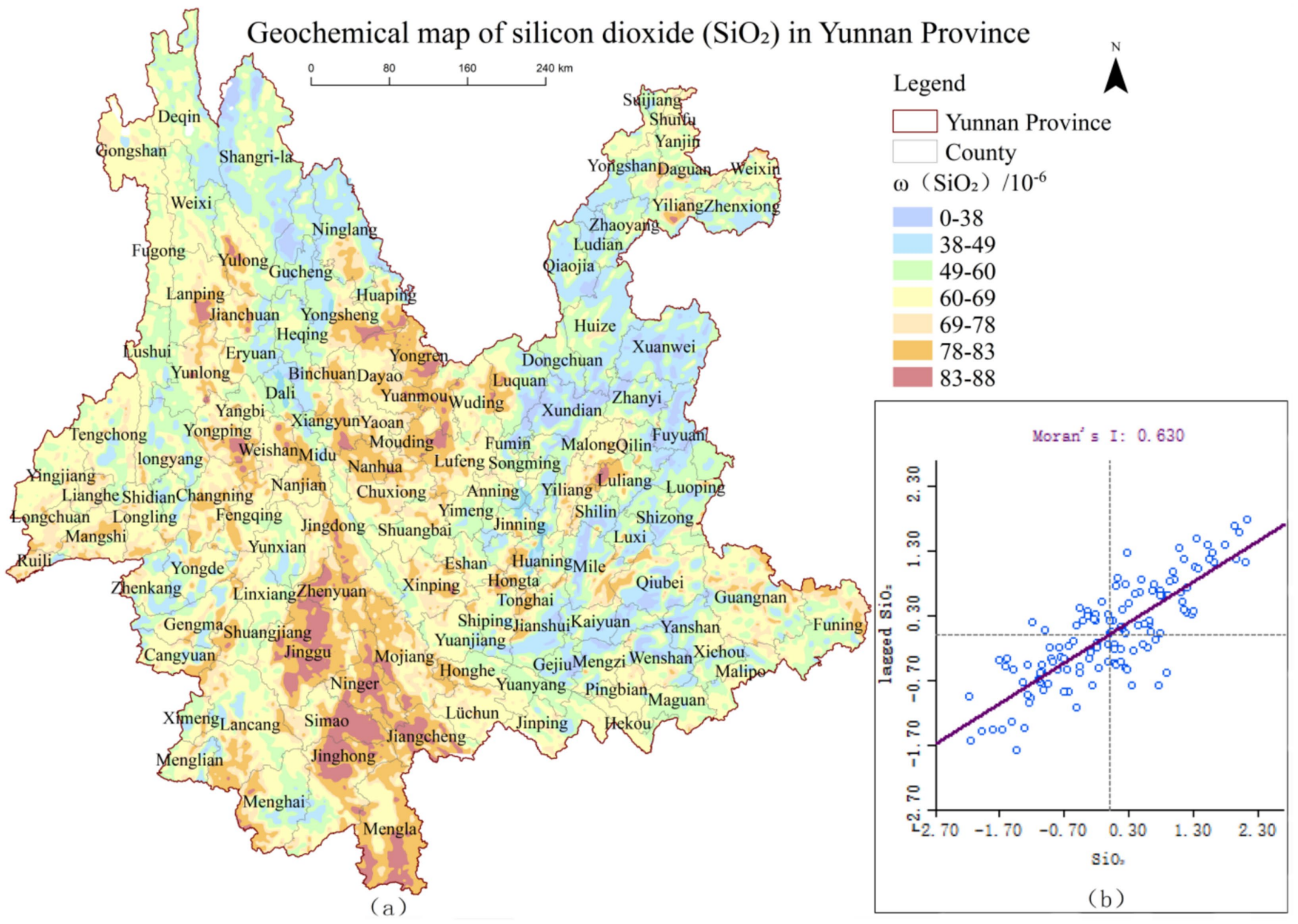
Spatial distribution pattern of *ω*(SiO_2_) and global autocorrelation scatter plot in Yunnan Province.

Using ArcGIS 10.8, the geochemical maps of MgO and SiO₂ from the Geophysical and Geochemical Map of Yunnan were vectorized and converted into raster data to produce spatial distribution pattern maps of *ω*(MgO)and *ω*(SiO₂), shown in Figures 2a, 3a, respectively. To further investigate the spatial distribution characteristics of *ω*(MgO) and *ω*(SiO₂) at the county level in Yunnan Province, global spatial autocorrelation analysis was conducted, and scatter plots were visualized using GeoDa software. The results are presented in Figures 2b, 3b.

As illustrated in Figures 2b, 3b, the Moran’s *I* indices for *ω*(MgO) and *ω*(SiO₂) are 0.474 and 0.630, respectively, with most data points located in the first and third quadrants. This indicates a predominance of high-high and low-low clusters in terms of their spatial distribution. Further randomization analysis of the results revealed that the Moran’s *I* indices of *ω*(MgO) and *ω*(SiO₂) exceeded their respective expected indices, with *p*-values less than 0.05 and Z-scores greater than 1.96. These findings indicate significant positive spatial autocorrelation for MgO and SiO₂ distributions at the county level in Yunnan Province, suggesting clear spatial clustering patterns rather than random distribution. Additionally, the Moran’s *I* index of *ω*(SiO₂) is higher than that of *ω*(MgO), indicating that SiO₂ exhibits a stronger spatial clustering tendency compared to MgO.

#### 3.1.2 Spatial distribution patterns of *ω*(MgO) and *ω*(SiO₂)

To comprehensively identify the spatial correlation patterns of *ω*(MgO) and *ω*(SiO₂), particularly the local association characteristics among data points, a cluster and outlier analysis was conducted at the county level in Yunnan Province. The results, as shown in Table 1, indicate that the spatial distribution patterns of MgO and SiO₂ can be categorized into four types: high-high clusters (HH), high-low outliers (HL), low-high outliers (LH), and low-low clusters (LL). Among these, low-value clusters (LL) and high-value clusters (HH) account for a significant proportion, reflecting notable spatial aggregation. In contrast, high-low (HL) and low-high (LH) outlier patterns occur less frequently, suggesting that spatial anomalies are relatively rare in the distribution.

**Table 1:** Scale of distribution patterns of *ω*(MgO), *ω*(SiO_2_).

*ω*(oxide)	HH		HL		LH		LL	
	Count	%	Count	%	Count	%	Count	%
*ω*(MgO)	13	10.07	1	0.77	2	1.55	12	9.30
*ω*(SiO_2_)	20	15.50	4	3.10	1	0.77	25	19.37

**Figure 4:**
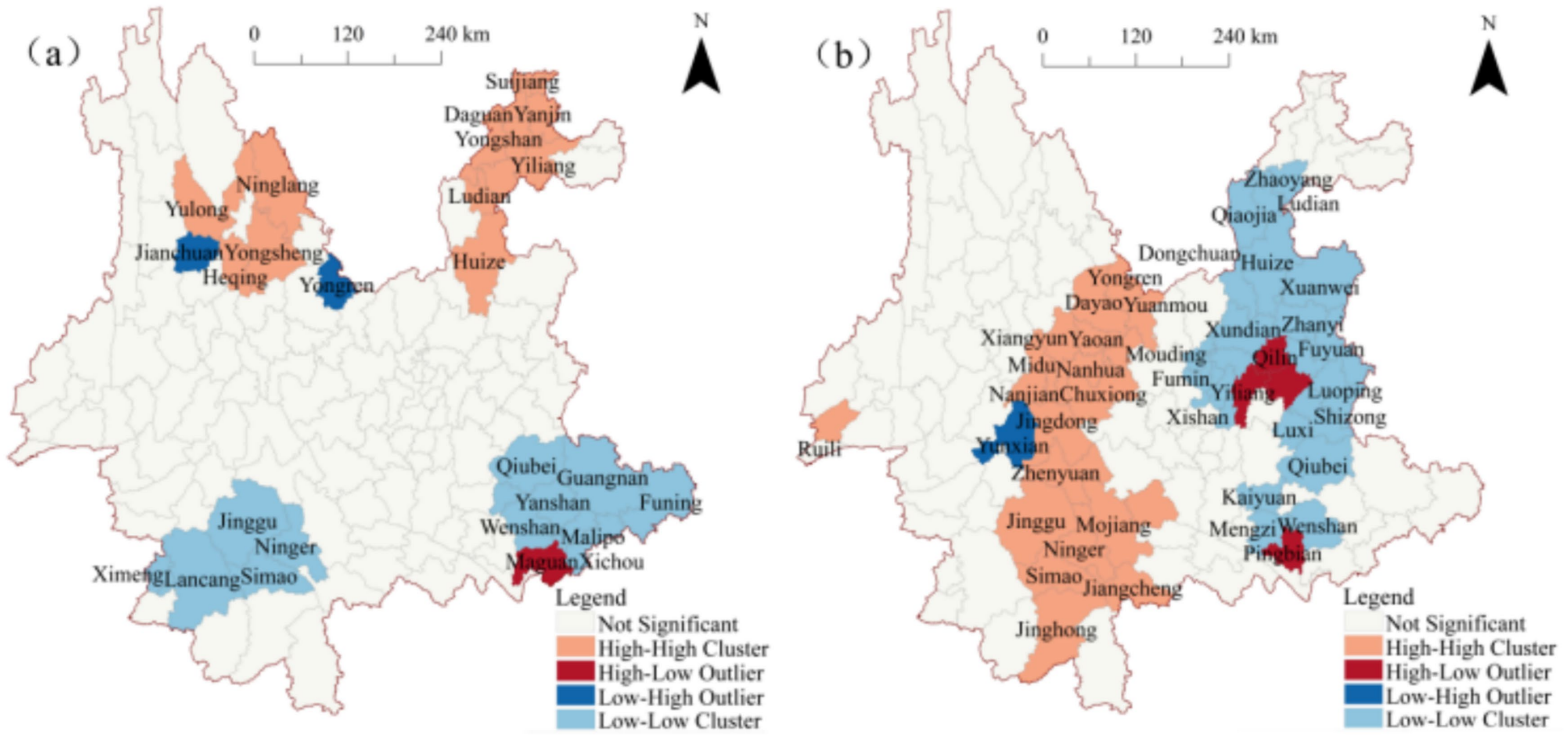
Local spatial autocorrelation maps of *ω*(oxide) in Yunnan Province **(a)** *ω*(MgO); **(b)** *ω*(SiO₂).

To further clarify the clustered regions and their spatial distribution characteristics, the results of the cluster and outlier analysis were visualized using ArcGIS, as shown in Figure 4. As depicted in Figure 4a, at the county level in Yunnan Province, high-value clusters of *ω*(MgO) are primarily concentrated in the northwestern and northeastern regions, specifically in 13 counties including Yulong Naxi Autonomous County, Heqing County, and Huize County. Low-value clusters, on the other hand, are mainly distributed in the southwestern and southeastern regions, encompassing 12 counties such as Lancang Lahu Autonomous County, Ning’er Hani and Yi Autonomous County, Yanshan County, and Qiubei County. Additionally, low-high (LH) outlier clusters are located in Jianchuan and Yongren Counties in the northwestern region, while high-low (HL) outlier clusters are concentrated in Maguan County in the southeastern region.

Figure 4b illustrates that high-value clusters of *ω*(SiO₂) are predominantly distributed in the central-western and southwestern regions of Yunnan, primarily within Chuxiong Prefecture and Pu′er City, covering 20 counties such as Yongren County, Yuanmou County, Menghai County, Jiangcheng, Hani and Yi Autonomous County. Conversely, low-value clusters are concentrated in the eastern region of Yunnan, with Kunming and Qujing as the core areas, radiating toward the northern part of Honghe Prefecture and the southern part of Zhaotong City. These low-value clusters include 25 counties such as Xishan District, Chenggong District, Songming County, Fuyuan County, and Luoping County. High-low (HL) outlier clusters are found in the central-eastern and southwestern regions, including Yiliang County, Luliang County, Malong District, and Pingbian Miao Autonomous County, surrounded by low-value clusters. Meanwhile, low-high (LH) outlier clusters are located in Yun County in the western region.

Overall, the spatial distribution patterns of *ω*(SiO₂) and *ω*(MgO) exhibit limited spatial correlation, with overlap observed only in high-value clusters within Wenshan City. This suggests that the clustering characteristics of *ω*(SiO₂) and *ω*(MgO) are largely independent, with minimal spatial overlap.

#### 3.1.3 Hotspot detection of *ω*(MgO) and *ω*(SiO₂) distribution

The cluster and outlier analysis revealed the local clustering and anomaly distribution characteristics of *ω*(MgO) and *ω*(SiO₂). Based on this, a spatial distribution framework was constructed from the local to the global scale. GIS was further employed to conduct hotspot analysis of *ω*(MgO) and *ω*(SiO₂) at the county level in Yunnan Province, aiming to identify high-density distribution regions at the regional scale. The results are presented in Table 2.

**Table 2:** Hot spot analysis results of *ω*(MgO) and *ω*(SiO_2_).

*ω*(oxide)	Positive hot spot		Negative hot spot	
	Count	%	Count	%
*ω*(MgO)	19	14.72	13	10.07
*ω*(SiO_2_)	24	18.60	32	24.80

The analysis shows that the significance levels of *ω*(MgO) and *ω*(SiO₂) are both greater than 0.05, allowing the identification of positive and negative hotspots, representing counties with relatively high or low concentrations, respectively. As shown in Table 2, the number of hotspot regions for *ω*(SiO₂) is significantly greater than that for *ω*(MgO). The proportions of positive hotspots for both indicators are relatively close, accounting for 14.72 and 18.60%, respectively. However, there is a notable difference in the proportion of negative hotspots: *ω*(SiO₂) has a negative hotspot proportion of 24.80%, which is significantly higher than the 10.07% for *ω*(MgO). These results indicate that the extreme spatial distribution of *ω*(SiO₂) (including high-value and low-value regions) is more pronounced than that of *ω*(MgO), particularly in terms of the spatial aggregation of low-value regions.

**Figure 5:**
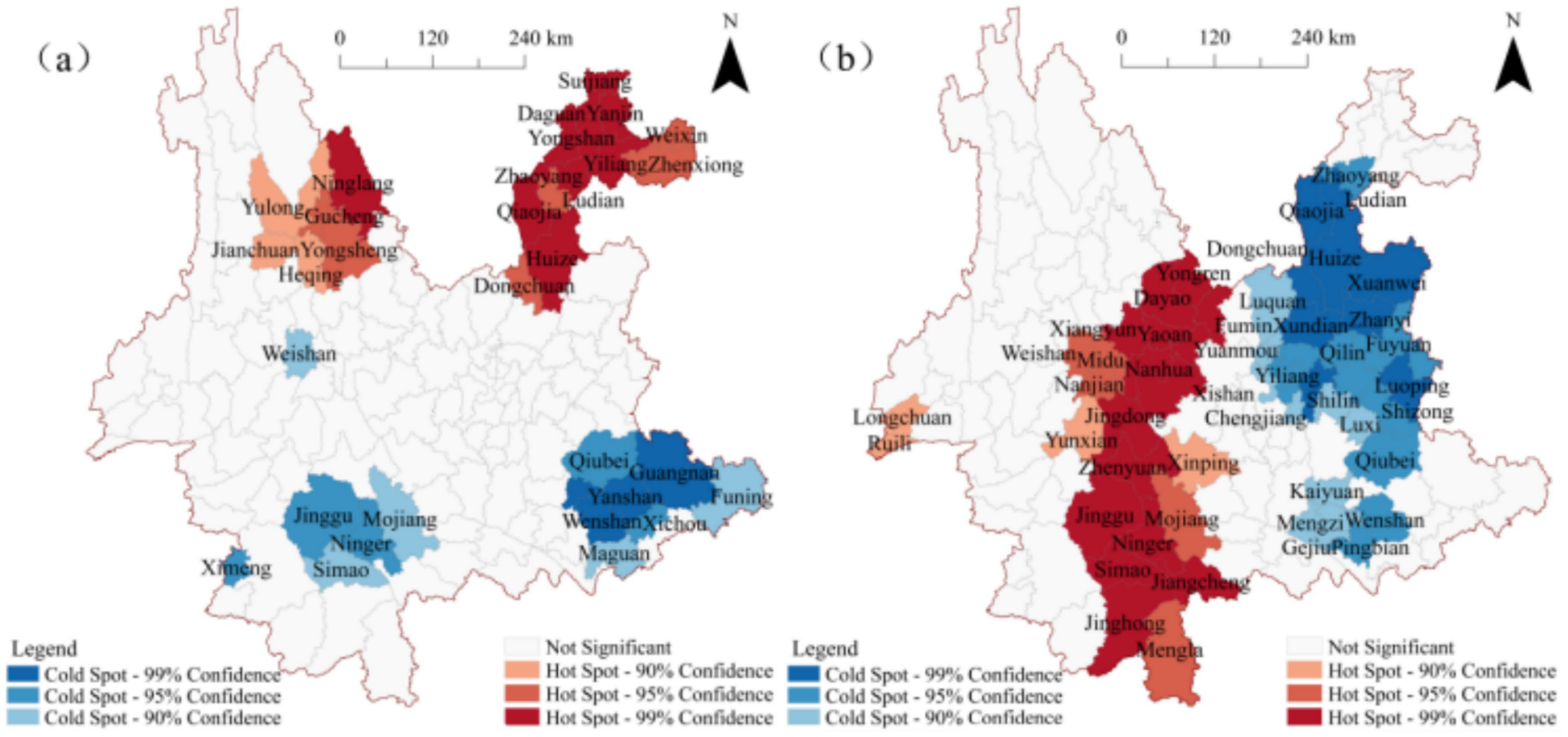
Spatial distribution of hotspot regions for *ω*(oxide) in Yunnan Province **(a)** *ω*(MgO); **(b)** *ω*(SiO₂).

To further visualize the spatial locations of the hotspot regions for *ω*(MgO) and *ω*(SiO₂), GIS was employed to generate spatial distribution maps, as shown in Figure 5. The maps reveal that positive hotspot regions of *ω*(MgO) are primarily distributed in the northwestern and northeastern counties of Yunnan, while those of *ω*(SiO₂) are concentrated in the central and southwestern counties. This indicates a clear spatial differentiation between the two in their distribution patterns. For negative hotspot regions, overlap is observed between *ω*(MgO) and *ω*(SiO₂) in Wenshan City, identifying it as a shared low-value enrichment area for both components. Combining this observation with the results of the cluster and outlier analysis (Figure 4), it can be further noted that most of the local high-value and low-value clustering regions are located within the significant positive and negative hotspot regions. This consistency suggests that the local spatial characteristics and overall trends mutually reinforce each other within the hotspot regions, enhancing the reliability of the spatial distribution patterns.

### 3.2 Correlation analysis between MgO, SiO₂, and population longevity

According to the findings of Wang Yu et al., the spatial distribution of population longevity levels in Yunnan Province exhibits significant clustering characteristics, with notable regional disparities at the county level. The distribution follows a “three-pole and multi-center” pattern, with the three major longevity regions primarily concentrated around Dianchi Lake, Nujiang River, and Honghe River (29). At the county level, high-value longevity clusters are mainly distributed in two regions. The first is the central Yunnan area, within Kunming City, including 10 counties such as Songming County, Xundian Hui and Yi Autonomous County, and Anning City. The second is the southeastern Yunnan region, located in the southeastern part of Honghe Prefecture and the southwestern part of Wenshan Prefecture, covering 8 counties such as Yuanyang County, Maguan County, and Wenshan City. In contrast, low-value clusters are primarily concentrated in the eastern part of Chuxiong Prefecture, the eastern region of Dali Prefecture, and the northern part of Pu′er City, encompassing 15 counties such as Dayao County, Yongren County, and Xiangyun County.

**Figure 6:**
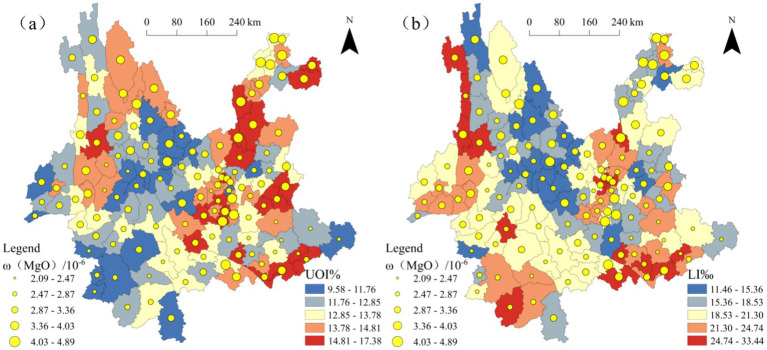
Overlay map of *ω*(MgO) and population longevity levels in Yunnan Province **(a)** *ω*(MgO) and UOI% **(b)** *ω*(MgO) and LI‰.

**Figure 7:**
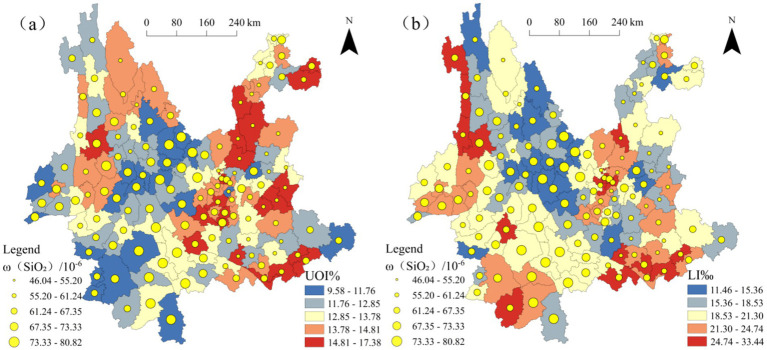
Overlay map of *ω*(MgO) and population longevity levels in Yunnan Province **(a)** *ω*(SiO₂) and UOI% **(b)** *ω*(SiO₂) and LI‰.

To explore the relationships between MgO, SiO₂, and population longevity levels, the natural breaks classification method was applied to categorize *ω*(MgO), *ω*(SiO₂), the Ultra-octogenarian Index, and the longevity index into five levels. Using the population longevity indicators, the Ultra-octogenarian Index and longevity index, as the baseline, *ω*(MgO) and *ω*(SiO₂) were overlaid to generate Figures 6, 7, respectively. Analysis of Figure 6a shows that, among the 51 counties with a higher Ultra-octogenarian Index range (13.78–17.38), 35 counties have *ω*(MgO) values greater than 2.87, of which 22 counties fall within the higher range (3.36–4.89). Similarly, Figure 6b indicates that, among the 41 counties with a higher longevity index range (21.30–33.44), 22 counties have *ω*(MgO) values greater than 2.87, with 8 counties falling within the higher range (3.36–4.89). Figure 7a shows that, among the 51 counties with a higher Ultra-octogenarian Index range (13.78–17.38), 42 counties have *ω*(SiO₂) values less than 67.35, of which 28 counties fall within the lower range (46.04–55.20). Figure 7b reveals that, among the 41 counties with a higher longevity index range (21.30–33.44), 30 counties have *ω*(SiO₂) values less than 67.35, with 20 counties falling within the lower range (46.04–55.20).

In summary, the spatial analysis indicates differing relationships between MgO, SiO₂, and regional population longevity levels. In regions with higher population longevity, *ω*(MgO) predominantly falls within higher value ranges, while *ω*(SiO₂) is predominantly within lower value ranges.

**Figure 8:**
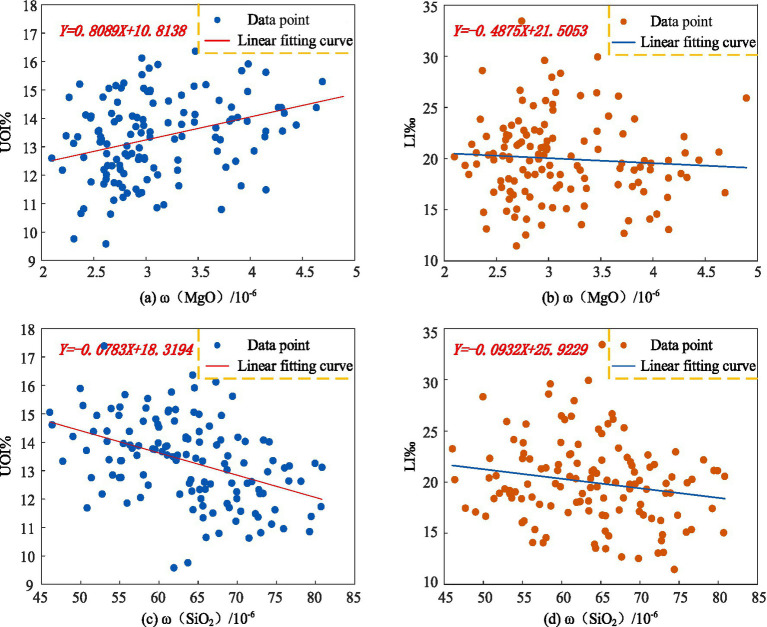
Scatter plots of correlations between *ω*(MgO), *ω*(SiO₂), and population longevity in Yunnan Province **(a)** *ω*(MgO) and UOI% **(b)** *ω*(MgO) and LI‰ **(c)** *ω*(SiO₂) and UOI% **(d)** *ω*(SiO₂) and LI‰.

To visually illustrate the correlations between MgO, SiO₂, and population longevity, bivariate scatter plots were generated using MATLAB, with curve fitting applied to the data. The results are shown in Figure 8. As depicted in Figures 8a,b, *ω*(MgO) exhibits a positive correlation with the Ultra-octogenarian Index and a negative correlation with the longevity index. Similarly, Figures 8c,d indicate that *ω*(SiO₂) demonstrates negative correlations with both the Ultra-octogenarian Index and the longevity index.

The scatter plots generated in MATLAB only illustrate the trends in relationships between the sample variables in this study. To determine the statistical correlations between the variables at the regional level, further significance testing is required. Using SPSS 27.0 software, a bivariate analysis was conducted by applying Pearson’s correlation coefficient to examine the relationships between *ω*(MgO), *ω*(SiO₂), and the longevity index and Ultra-octogenarian Index at the county level in Yunnan Province. The significance of these correlations was also tested, with the results presented in Table 3. As shown in Table 3, *ω*(MgO) exhibits a significant correlation only with the Ultra-octogenarian Index. In contrast, *ω*(SiO₂) shows negative correlations with both the longevity index and the Ultra-octogenarian Index, and both correlations pass the significance test.

**Table 3:** Correlation analysis between *ω*(MgO), *ω*(SiO₂), an population longevity indicators.

*ω*(oxide)	Statistical Indicators	The longevity index	The ultra-octogenarian Index
*ω*(MgO)	r	−0.070	0.308**
	Sig.	0.429	<0.001
	N	129	129
*ω*(SiO_2_)	r	−0.174*	−0.463**
	Sig.	0.049	<0.001
	N	129	129

**Indicates significance at the 0.01 confidence level; *indicates significance at the 0.05 confidence level.

Although *ω*(MgO) is not correlated with the longevity index, it demonstrates a positive correlation with the Ultra-octogenarian Index, with a correlation coefficient of 0.308, passing the significance test at the 0.01 confidence level. His finding suggests a correlation between the MgO geochemical background value and the older adult population level at the county scale in Yunnan Province. In contrast, *ω*(SiO_2_) exhibits a negative correlation with both the longevity index and the Ultra-octogenarian Index, with correlation coefficients of −0.174 and −0.463, respectively. These relationships are significant at the 0.05 and 0.01 confidence levels, respectively, suggesting a negative correlation between the SiO_2_ geochemical background value and the population’s longevity at the county level in Yunnan Province.

### 3.3 The synergistic effect of MgO and SiO₂ on regional longevity levels

In this study, the relationships between MgO, SiO₂, and regional population longevity levels were analyzed individually. The results reveal a positive association between MgO and regional longevity levels, while SiO₂ shows a negative correlation with longevity. Although the independent relationships of these two oxides with longevity have been identified, MgO and SiO₂ often coexist in natural soil environments. Therefore, it is essential to further explore their synergistic effects on regional longevity levels.

**Figure 9:**
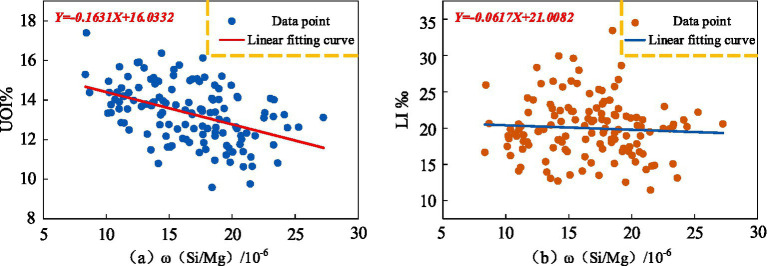
Scatter plots of the correlation between *ω*(Si/Mg) and population longevity **(a)** *ω*(Si/Mg) and UOI% **(b)** *ω*(Si/Mg) and LI‰.

Using the geochemical background values of MgO and SiO₂ the ratio of Mg to Si was calculated, represented as *ω*(Si/Mg), to quantify the synergistic effect of MgO and SiO₂. This ratio was used as the independent variable. Scatter plots were generated, and curve fitting was performed using MATLAB to visually illustrate the relationship between the synergistic effects of these elements and regional population longevity levels. The results are shown in Figure 9. Figure 9a demonstrates a negative correlation between *ω*(Si/Mg) and the Ultra-octogenarian Index. Similarly, Figure 9b indicates a negative correlation between *ω*(Si/Mg) and the longevity index, although the correlation is relatively weaker. These findings provide insights into the complex interplay between MgO and SiO₂ and their combined influence on regional longevity levels.

The scatter plots illustrate a negative correlation between the synergistic effect of *ω*(Si/Mg) and both the Ultra-octogenarian Index and the longevity index. To further confirm the relationship between the synergistic effect and regional population longevity at the statistical level, significance tests were performed. The results are presented in Table 4. As shown in Table 4, the correlation indices between *ω*(Si/Mg) and both the longevity index and Ultra-octogenarian Index are negative. Specifically, the negative correlation with the Ultra-octogenarian Index is statistically significant, whereas the correlation with the longevity index does not pass the significance test.

**Table 4:** Correlation analysis between the synergistic effect of *ω*(MgO) and *ω*(SiO₂) and population longevity indicators.

*ω*(specific value)	Statistical Indicators	The longevity index	The ultra-octogenarian Index
*ω*(Si/Mg)	r	−0.042	−0.459**
	Sig.	0.637	<0.001
	N	129	129

**Indicates significance at the 0.01 confidence level; *Indicates significance at the 0.05 confidence level.

Although no significant statistical correlation was found between *ω*(Si/Mg) and the longevity index in the current study, the correlation coefficient between *ω*(Si/Mg) and the Ultra-octogenarian Index was −0.459, which passed the significance test at a confidence level of 0.01. This finding suggests a non-negligible relationship between the synergistic effects of MgO and SiO₂ and regional population longevity levels.

## 4 Discussion

Yunnan Province, characterized by geological and geomorphological diversity, ecological variety, diverse soil types, and significant differences in resource development, economic progress, and healthcare levels, exhibits a clear statistical pattern. Specifically, there is a significant positive correlation between *ω*(MgO) and the Ultra-octogenarian Index, as well as significant negative correlations between *ω*(SiO_2_) and both the longevity index and the Ultra-octogenarian Index. Additionally, a significant negative correlation is observed between *ω*(Si/Mg) and the Ultra-octogenarian Index. The underlying factors driving these relationships merit further exploration.

Although longevity does not equate to health, numerous studies suggest that individuals in longevity populations typically maintain good health for the majority of their lives. Maintaining good health is a key determinant of longevity, and longevity is also trending towards being healthier (40–42). MgO in soil is absorbed by plant root systems and transferred to humans through the biological food chain. Magnesium is crucial for stabilizing the double-helix structure of DNA and RNA and ensuring the proper functioning of metabolic processes in the human body. In aquatic systems, MgO can lower fluoride concentrations and release magnesium ions, which enhance disease resistance and prevent fluorosis (43). Studies have also shown an inverse relationship between magnesium concentrations in soil and drinking water and cardiovascular disease mortality rates (44, 45). MgO is widely used as a food additive, color stabilizer, and pH regulator, providing a magnesium supplement in health products and food. In agriculture, it is utilized as a magnesium fertilizer to prevent magnesium deficiency in plants. Additionally, it is added to livestock feed to enhance the quality of meat, eggs, milk, and other agricultural products. As a basic oxide, MgO can also mitigate the release of harmful acidic gases like hydrogen sulfide (H₂S) in the environment (46), thereby improving living conditions and indirectly contributing to human health and longevity.

This study reveals that although silicon (Si) is an essential trace element for the human body, it exhibits a negative correlation with regional longevity levels. One possible explanation is that excessive SiO₂ intake can have adverse effects on renal health, potentially leading to the development of kidney diseases (47). Additionally, in regions with high SiO₂ background values, SiO₂ may readily enter the atmosphere, forming fine inhalable SiO₂ particles (48). The inhalation of crystalline SiO₂ is associated with the development of silicosis, a progressive fibrotic lung disease, and prolonged respiratory exposure can increase the risk of cancer (49). Additionally, natural SiNPs (nano-SiO_2_) are also recognized as a significant source of atmospheric inorganic particulate pollution, which can lead to pulmonary diseases (50). This is one of the major causes of the higher mortality rates in these regions, thus affecting the longevity levels to some extent. Previous studies have indicated that the high incidence of lung cancer among women in Xuanwei City, within the study area, is associated with the presence of ultrafine SiO_2_ in the coal mined and used locally (51).

The synergistic interaction between MgO and SiO_2_ plays a crucial role in shaping the longevity levels of regional populations. Specifically, an increase in the *ω*(Si/Mg) value, which signifies a higher relative concentration of SiO_2_ compared to MgO in the environment, is associated with a decline in the population’s longevity. This can be attributed to several factors. First, Si and Mg exhibit a competitive relationship (52). Elevated Si concentrations in the soil may reduce the bioavailability of Mg, thereby limiting the absorption of Mg by plants (53), which in turn affects human magnesium intake. Secondly, the ratio of MgO to SiO_2_ also influences the soil pH. As an acidic oxide, the concentration of SiO_2_ in the soils of Yunnan Province significantly exceeds that of the basic oxide MgO, leading to changes in soil pH. When the soil pH is low, essential nutrients such as phosphorus (P), potassium (K), and magnesium (Mg) become less soluble, making it difficult for plants to absorb these nutrients (54). This, in turn, impacts the intake of essential elements by humans. Therefore, maintaining an optimal ratio of MgO to SiO_2_ in the soil may have significant potential implications for regional population longevity.

This study has several limitations. Although it provides valuable insights into regional-level relationships that can inform local policies for promoting longevity tourism or health initiatives, it does not offer a precise explanation of the causal links between individual longevity and other confounding factors at the personal level. Future research should integrate genetic factors, healthcare access, lifestyle choices, socioeconomic status, altitude, pollution, and other relevant variables to better understand their relationship with longevity. Furthermore, this study is based on a cross-sectional design, which limits its ability to capture dynamic changes over time. Future studies should adopt a longitudinal approach to strengthen the temporal depth of the research.

## 5 Conclusion

Although the geochemical background values of MgO and SiO₂ do not directly correspond to the amounts that can be absorbed by individuals, research at the county scale indicates that these values are spatially associated with regional population longevity phenomena. The specific conclusions are as follows:

(1) At the county scale in Yunnan Province, the geochemical background value of MgO exhibits a positive correlation with the Ultra-octogenarian Index. This suggests that MgO may positively influence the longevity of the regional population. This finding underscores the potential importance of MgO content in maintaining and promoting human health and longevity.
(2) At the county scale in Yunnan Province, the geochemical background value of SiO₂ demonstrates a negative correlation with regional population longevity. Statistical analysis reveals significant negative correlations between the geochemical background value of SiO₂ and both the longevity index and the Ultra-octogenarian Index, indicating that SiO₂ content may have a detrimental effect on the longevity of the regional population.
(3) The analysis of the synergistic effects of MgO and SiO₂ on regional population longevity levels reveals a significant negative correlation between *ω*(Si/Mg) and the Ultra-octogenarian Index. This suggests that the interaction between MgO and SiO₂ in the environment may influence the availability of essential nutrients, the extent of plant nutrient absorption, and soil pH, thereby indirectly affecting the intake of essential elements by humans and, ultimately, impacting regional longevity. In conclusion, maintaining an appropriate ratio of MgO to SiO₂ in the soil is crucial for promoting regional population longevity.

## Data Availability

The original contributions presented in the study are included in the article/supplementary material, further inquiries can be directed to the corresponding author.
